# Narrative warmth and quantitative competence: Message type affects impressions of a speaker

**DOI:** 10.1371/journal.pone.0226713

**Published:** 2019-12-23

**Authors:** Jenna L. Clark, Melanie C. Green, Joseph J. P. Simons

**Affiliations:** 1 Center for Advanced Hindsight, Duke University, Durham, North Carolina, United States of America; 2 Department of Communication, University at Buffalo, Buffalo, New York, United States of America; 3 Department of Psychology, University of North Carolina at Chapel Hill, Chapel Hill, North Carolina, United States of America; 4 Institute of High Performance Computing, Singapore, Singapore; West Virginia University, UNITED STATES

## Abstract

Persuasion research often focuses on how source characteristics affect attitude change in response to a message; however, message characteristics may also alter perceptions of the source. The Message-Based Impression Formation effect (M-BIF) suggests that perceivers use features of messages to infer characteristics of the source, and that such inferences may have a variety of consequential outcomes. In particular, the choice of narrative versus statistical evidence may have implications for the perceived warmth and competence of a source. In five experiments, narrative arguments led to greater perceptions of source warmth and statistical arguments led to greater perceptions of source competence. Across the two behavioral studies, a matching effect emerged: participants preferred to work on cooperative tasks with partners who had provided narratives, and competitive tasks with partners who had provided statistical evidence. These results suggest that the evidence type chosen for everyday communications may affect person perception and interpersonal interaction.

## Introduction

Imagine you are on a blind date with a friend of a friend. You sit across a restaurant table from your prospective romantic partner, both smiling at the awkwardness of the situation. Trying to break the ice, you ask about their summer travel plans. With obvious relish, they whip out their smartphone and start explaining the complex cost-benefit analysis by which they planned an upcoming holiday. Immediately, you feel you know something more about the kind of person with whom you are having dinner.

**Fig 1:**
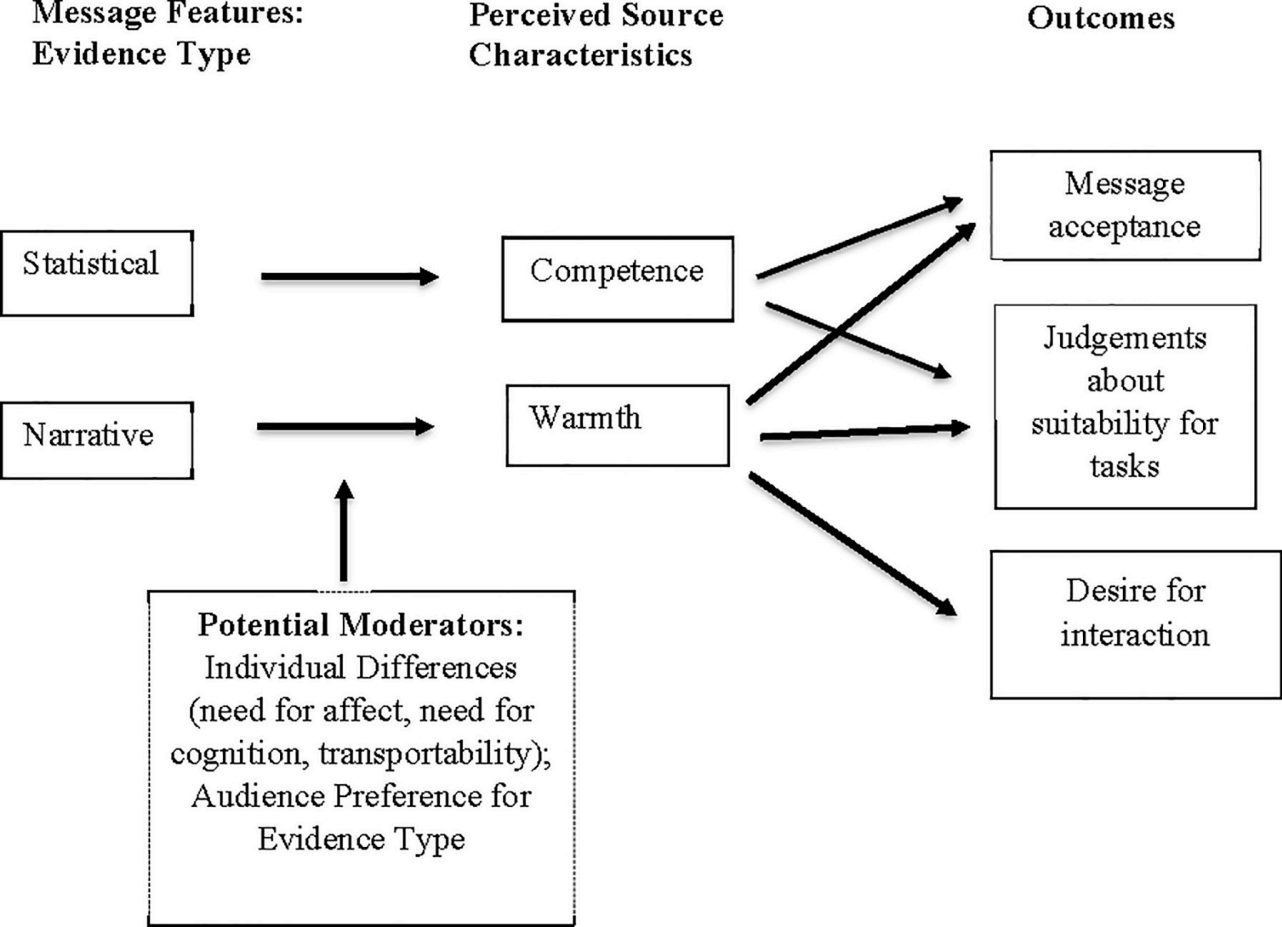
Message-based impression formation effect.

The (somewhat whimsical) example above illustrates what we term the Message-Based Impression Formation effect (M-BIF; see Fig 1). The core idea is simply that the kind of arguments someone makes affects how other people perceive them. Person perception has usually been emphasized as an input to the persuasive process–people are more likely to believe someone trustworthy and competent. However, we point to the fact it can also be an outcome–the way someone argues communicates information about them.

Here, we focus on how the evidence type used in a persuasive appeal can affect impressions of the message source (speaker). Specifically, we draw a distinction between two broad types of evidence: narrative and statistical. This distinction between different modes of argument has been explored by Bruner [1], and loosely maps on to Aristotle’s distinction between pathos (emotional) and logos (logical). As will be discussed in more detail below, we propose that narrative appeals convey an impression of interpersonal warmth whereas statistical arguments connote competence.

Evidence type was chosen as we judged it a particularly promising argument feature. It is intuitively plausible that it affects person perception, the two argument types map neatly onto well-validated impression formation models, and findings on this question have clear implications for how experts can contribute to public discourse. Our focus on evidence type does not preclude that other features also affect impression formation. Other prominent examples include clarity and organization (which could convey conscientiousness, for example), specific word choice (e.g. affectively positive vs. negative language), and gain vs. loss framing. Of course, many other aspects of persuasive appeals likely also affect impression formation. We focus on the effects of evidence type because it is intuitively plausible, maps message dimensions clearly onto a well-validated impression formation model, and has implications for the best ways for experts to contribute to public discourse.

### Person perception and source factors: Warmth / competence and credibility / liking

Perceptions of people revolve around two dimensions: warmth and competence [2,3]. Fiske, Cuddy, and Glick [4] define warmth as “perceived intent: friendliness, sincerity, helpfulness, trustworthiness and morality” and competence as “perceived ability: intelligence, creativity, efficacy, and skill” (p. 77). Other terms such as dominance and friendliness [5] and agency and communion [6], have also been used to conceptualize the divide, but are largely synonymous. These perceptions have consequences across a range of interpersonal domains, from discrimination [4] to consumer behavior [7].

Although our focus here is on person perception, the competence and warmth dimensions in impression formation parallel source factors in traditional persuasion research–in particular, the crucial source factors of credibility and liking. Credibility is defined typically as a combination of expertise [8] and trustworthiness [9]; in other words, a credible communicator is one who knows the topic well and lacks ulterior motives. Greater credibility tends to lead to greater persuasion [9], and–given its link with expertise–likely correlates with competence. Liking also promotes social influence [10], and may arise from pure physical attractiveness [11, 12] similarity [13], and familiarity [14]. As warmth is typically defined with concepts such as friendliness, sincerity, and helpfulness, individuals who are liked are almost certainly seen as high in warmth. Links between person perception and source factors therefore seem a natural fit: credibility and competence both stem from perceptions of knowledge and ability, whereas liking and perceived warmth both stem from positive affective reactions.

Liking/warmth and expertise/competence are typically manipulated by altering obvious aspects of the source, such as physical appearance or job title, and then observing if these manipulations affect persuasion. Our studies of the Message-Based Impression Formation effect reverse the traditional emphasis by treating warmth and competence as dependent variables, to be inferred from the persuasive message itself. For example, an argument that is heavy on impressive detail may imply expertise above and beyond any title given to the source.

Our research is not the first to examine the effects of messages on perceptions of the source. For example, research on the attributional analysis of persuasion suggests that whether or not sources confirm the expectancies audiences have of them can influence perceptions of that source’s credibility (e.g., sources that argue against their own interests may appear particularly honest or credible [15, 16]). However, the influence of messages on sources remains a relatively understudied area.

### Statistical and narrative evidence in persuasion

Persuasive messages may rely on either narrative or statistical evidence to make their case. For example, one person may try to convince another about the benefits of a new medical treatment with a touching story of a successful cure, while the other person may counter that research shows the treatment is no better than a placebo. Narrative arguments typically provide the story of an individual; Kreuter et al. [17] define a narrative as “a representation of connected events and characters that has an identifiable structure, is bounded in space and time, and contains implicit or explicit messages about the topic being addressed” (p. 222). Statistical arguments focus on providing factual information, often incorporating numerical data and summarizing the experience of multiple people. For example, in promoting a preventative health program, a statistical argument might provide numerical information about the benefits of the program. A narrative argument might tell the story of an individual who followed the program, clearly linking their actions to positive consequences.

Prior research concentrated on the question of whether narrative or statistical persuasion is more effective. The results have been mixed. Reviews [18, 19] found that narrative information was more persuasive than statistical information. However, experimental work [20] and a meta-analysis [21] claimed the opposite.

More recent work seeks to explain these discrepancies by suggesting that that the two message types affect beliefs by different casual pathways. For example, statistical evidence produces greater cognitive effects (thoughts about the subject and message ratings), while narrative evidence produces greater affective effects (positive and negative emotional reactions) [22, 23]. Statistical evidence is more effective in altering beliefs and attitudes, which are arguably more cognitive in nature, while narrative evidence is more effective in altering intentions, which are more affective [24]. Additionally, several studies suggest that narrative evidence may be less cognitively elaborated, as it is subject to less defensive processing [25] and less counter-argument [26].

The mapping of statistical and narrative evidence onto cognitive and affective processing is highly suggestive of connections with person perception. Statistical messages use facts to change beliefs about the world, consistent with a message generator who is well informed and bases their decisions on beliefs. Narrative messages, however, generate sympathetic effects in the listener, consistent with a message generator who is attuned to the feelings of others. To put this in person perception terms, statistical messages are a cue to competence, whereas narrative messages are a cue to warmth.

### Evidence type and person perception

We propose that there are connections between competence, cognition, and statistical evidence, and between warmth, affect, and narrative evidence. More specifically, we propose two hypotheses:

**H1:** An individual who presents narrative evidence will be seen as warmer.
**H2**: An individual who presents statistical evidence will be seen as more competent.

Studies 1a and 1b investigate these initial hypotheses and demonstrate the basic effect of evidence type on person perception across two different populations. Study 2 replicates the effect while examining the potential moderating role of the target’s preference for an evidence type (that is, whether the recipient prefers to hear a story versus statistics). Studies 3a and 3b further extend the findings to demonstrate that evidence type also has meaningful behavioral effects.

### Individual differences

It is possible that audience factors (personality or information preferences) may moderate the effect of message features on impressions. In particular, our current studies also examine whether the hypothesized effects are moderated by individual differences: specifically, need for cognition (enjoyment of thinking) and need for affect (the tendency to seek out and enjoy emotional experiences). We also examined transportability (the tendency to become immersed in stories) and, in Study 2, subjective numeracy (comfort with numerical information). These variables were chosen based on the previously established links between statistics and cognition and narrative and affect, as well as the importance of transportability in narrative experience [27].

Although we expected the effects of evidence type to be robust, some previous research has suggested that individuals high in need for cognition are more sensitive to competence-relevant information, whereas individuals high in need for affect are more sensitive to warmth-relevant information [28]. Thus, high need for cognition individuals might show stronger effects for the statistics/competence relationship, and high need for affect individuals might show a stronger narrative/warmth relationship. Similarly, high transportability (the tendency to become immersed in stories) might show stronger effects for the narrative/warmth relationship, as such individuals are more influenced by narrative information generally, and highly numerate individuals might show stronger effects for the statistics/competence relationship. Because individuals’ perception of their numerical/mathematical abilities (subjective numeracy) is highly correlated with actual numeracy, is predictive of responses to risk communications, and is shorter and less stressful for participants, we examined subjective numeracy instead of objective [29].

**RQ1**: Are the effects of evidence type on person perception moderated by individual differences?

## Pretesting

To investigate our hypotheses, we tested specific arguments on financial, education, and weight-loss decisions to ensure that they did not systematically differ in quality between statistical and narrative conditions, and that the nature of each argument as statistical or narrative was clear. We did not attempt to change attitudes with these persuasive appeals. As such, passages were allowed to vary in content to ensure they read as naturalistic dialogue that was equal in quality between conditions.

### Method

#### Participants

Amazon Mechanical Turkers (N = 75) participated in pre-testing for small financial rewards. Amazon Mechanical Turk is an online service that allows individuals to pay people for completing specific tasks. It has been widely used for communication and psychological studies [30].

#### Procedure

Each participant rated one argument from each domain, counterbalanced for order. After reading an argument, participants rated it on several dimensions, using a scale from 1 (*not at all*) to 7 (*very much*). These questions examined relevance, persuasiveness, quality (“how good is the advice”) and the likelihood of the individual following the advice. All questions except relevance (due to lowered reliability) were combined in an aggregate quality measure with good reliability (Cronbach’s alphas = 0.85–0.91). Participants were also asked the extent to which the argument seemed to be based on “research or statistics” and “personal experience or stories” on the same 1–7 scale.

All studies reported in this manuscript were approved by the IRB of the University of North Carolina at Chapel Hill, and in all studies, participants read and agreed to an electronic consent form before beginning the study. Protocols and data for all studies are accessible at dx.doi.org/10.17504/protocols.io.7rahm2e.

### Results and discussion

The financial arguments did not differ significantly in quality between statistical and narrative conditions, *t*(72) = -1.22, *p* > .05, Cohen’s *d* = 0.27, nor did the education arguments, *t*(74) = -0.82, *p* > .05, *d* = 0.19; however, the narrative weight loss argument was significantly higher quality than the statistical argument, *t*(73) = 2.47, *p* = .02, *d* = 0.57. Across all three domains, statistical arguments were seen as significantly more statistical than narrative arguments, and narrative arguments were seen as significantly more narrative than statistical arguments. See Tables 1 and 2. The financial domain was chosen for subsequent studies.

**Table 1:** Quality judgments for arguments across domains.

*Finance*	*Mean*	*SD*	*df*	*t*	*d*
Narrative	5.25	1.24	71†	-1.22	0.27
Statistical	5.55	0.86			
*Education*	*Mean*	*SD*	*df*	*t*	*d*
Narrative	4.94	1.24	74	-0.82	0.19
Statistical	5.16	1.02			
*Weight Loss*	*Mean*	*SD*	*df*	*t*	*d*
Narrative	5.37	1.25	73	2.47*	0.57
Statistical	4.63	1.29			
*Learning*	*Mean*	*SD*	*df*	*t*	*d*
Narrative	4.7	1.55	101	-1.56	0.31
Statistical	5.13	1.21			

Note.

* = *p* < 0.05.

** = *p* < 0.01.

*** = *p* < 0.0001.

† = degrees of freedom modified due to significant Levene's test for equality of variances.

All data reported from full samples.

**Table 2:** Narrative and statistical character judgments for arguments across domains.

Domain	Narrative Character					Statistical Character				
*Finance*	*Mean*	*SD*	*df*	*t*	*d*	*Mean*	*SD*	*df*	*T*	*d*
Narrative	6.32	1.4	63†	6.74***	1.57	2.18	1.77	74	-8.79***	2.02
Statistical	3.67	1.96				5.42	1.4			
*Education*	*Mean*	*SD*	*df*	*t*		*Mean*	*SD*	*df*	*t*	
Narrative	6.35	1.18	63†	8.41***	1.88	1.88	1.45	71	-7.96***	1.87
Statistical	3.15	2.07				5.03	1.85			
*Weight Loss*	*Mean*	*SD*	*df*	*t*		*Mean*	*SD*	*df*	*t*	
Narrative	6.49	0.98	44†	8.23***	2.06	1.7	1.22	55†	-7.51***	1.79
Statistical	3.36	2				4.63	2.00			
*Learning*	*Mean*	*SD*	*df*	*t*		*Mean*	*SD*	*df*	*t*	
Narrative	6.32	1.21	85†	10.07***	2.01	2.21	1.57	102	-9.52***	1.87
Statistical	3.28	1.78				5.04	1.46			

Note.

* = *p* < 0.05.

** = *p* < 0.01.

*** = *p* < 0.0001.

† = degrees of freedom modified due to significant Levene's test for equality of variances.

All data reported from full samples.

## Study 1a

Study 1a was designed to test the basic effect of statistical versus narrative evidence on person perception, as well as providing an initial test of whether this effect was moderated by individual differences in need for cognition, need for affect, and transportability.

### Method

#### Participants

Undergraduates (N = 235) participated for partial course credit. The sample was majority female (66%) and white (70%). Ages ranged from 18 to 24 (*M* = 19.07, SD = 1.14).

For this study, the key effect is a two-way interaction between message type and personality dimension. We identified the smallest effect size we could reliably detect (80% power) with this sample size (using G*Power software [31]). This post-hoc sensitivity analysis shows our analysis is able to reliably detect even quite small effects (f2 = .03, *η*^2^ = .03).

#### Procedure

Participants accessed the survey online via Qualtrics. They were asked to read a vignette in which the target solicits advice on a topic and the source provides an argument that is either narrative or statistical, and rate this vignette on multiple dimensions. Participants then completed competence and warmth measures for the source, as well as individual difference measures.

Participants also read and rated the arguments on the weight loss domain, as mentioned in pre-testing; however, null results were found. We believe that the difference in quality between the narrative and statistical arguments represents a confound that cannot be separated from any effect of evidence type. As such, the weight loss domain is not discussed further or included in study 1b.

### Materials

#### Vignettes

The vignette began with an introduction that named two individuals, John (the asker, who had a question on a specific topic) and Dave (the source, a work acquaintance John would ask for advice). The asker then posed a question, and the source responded with either a narrative or statistical argument:

***John***: What do you think about me opening an account with First City Bank?
***Dave—Statistical Argument***: Well, First City Bank has a very good interest rate on their accounts compared to most banks, and they’ll also let you negotiate for an even better one. What else, let me think … I read an article on this the other day. Eighty percent of First City Bank’s customers report being satisfied with their banking, which is pretty much the highest rate around here.
***Dave—Narrative Argument***: Well, I have another friend who banked with them, and he said it was a great experience. When he wanted to buy a house, his banker gave him a choice between several different loans and took the time to explain each one very carefully. He was able to suggest one a program that got them a preferential rate, and my friend said it saved him thousands of dollars in the long run.

Both arguments ended with, “I think you ought to invest with them.”

#### Person perception

A modified version of Fiske, Cuddy, Glick, and Xu’s [32] Warmth and Competence Scale assessed person perception. For competence, participants rated the extent to which the source was competent, confident, independent, competitive and intelligent (5 items); for warmth, participants rated the extent to which the source was tolerant, good-natured, warm, sincere and friendly (5 items). All questions used a 1 (*not at all*) to 5 (*extremely*) scale.

#### Individual difference measures

Need for cognition, the tendency to engage in and enjoy cognitive effort, was measured with the 18-item Need for Cognition Scale [33]. Need for affect, the motivation to approach or avoid emotion-inducing situations, was measured with the 10-item Need for Affect Questionnaire–Short [34]. Transportability, the extent to which individuals typically become transported or immersed into stories, was measured with the 4-item Short Transportability Scale [23].

All studies also included measures of participants’ preferences for narrative and statistical information. These measures did not show consistent effects across studies or change the main pattern of results, and thus will not be discussed here.

#### Attention check

Participants were asked “What topic did the conversation you read discuss?” Participants passed this check if their answer related to banking or finance; four participants failed. No changes in significance occurred when these participants were excluded; therefore, all analyses are reported with the full sample.

### Results

**Fig 2:**
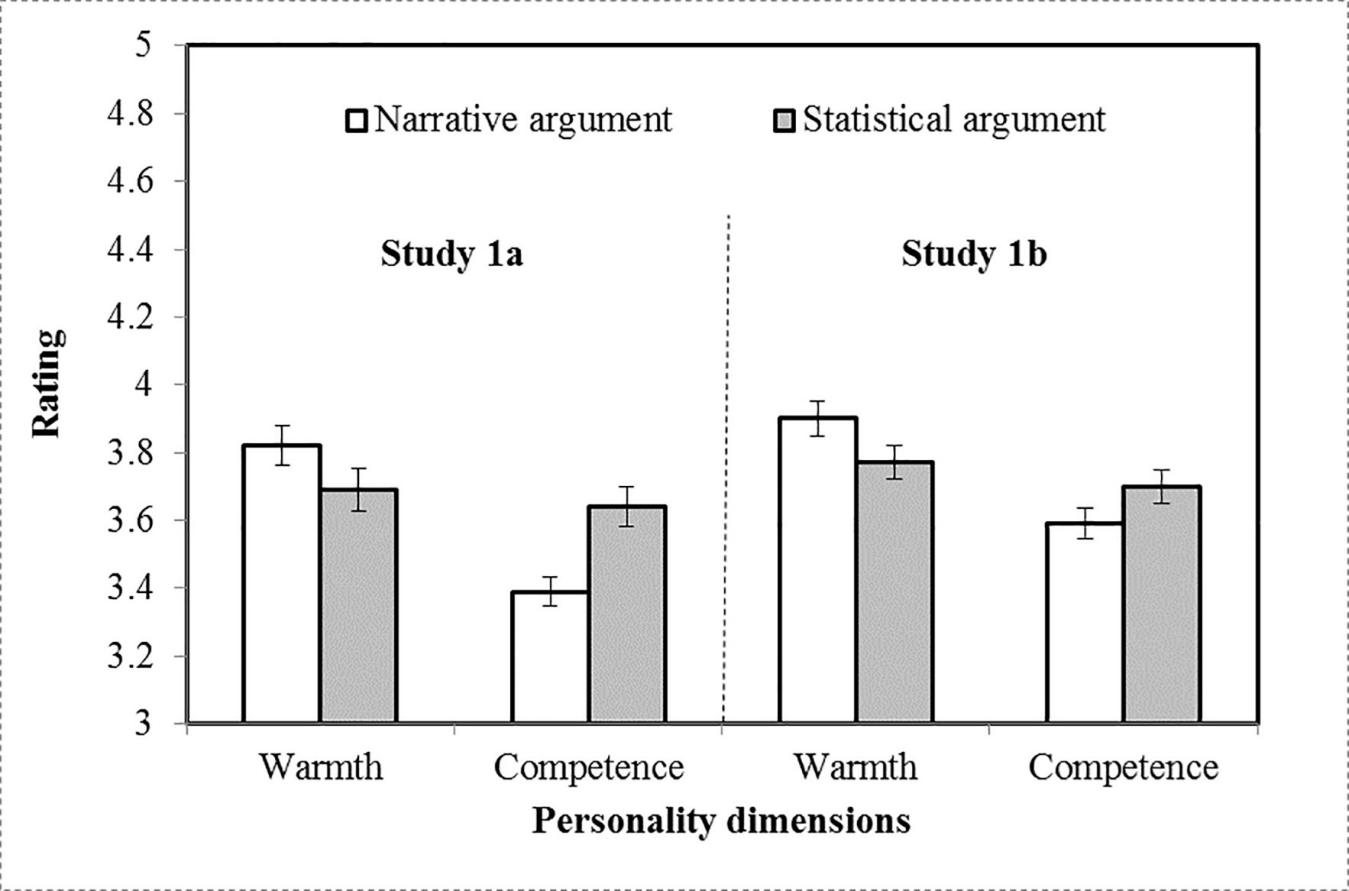
Mean warmth and competence ratings of a source using narrative vs. statistical argumentation across Studies 1a and 1b. *Note*. Error bars represent standard error.

As predicted, participants rated a source who used a narrative argument as warmer but less competent than a source who used a statistical argument (see Table 3 and Fig 2). A 2 (argument type: statistical vs. narrative) x 2 (rating: warmth vs. competence) ANOVA with repeated measures on the final factor showed this interaction effect was significant, F(1, 223) = 17.80, p < .001, η_*p*_^2^ = .07. Argument type had differential effects on the two person perception dimensions.

**Table 3:** Means of warmth and competence by evidence type and frame for studies 1–2.

Domain	Study 1a		Study 1b	
	*Statistics*	*Narrative*	*Statistics*	*Narrative*
Warmth	3.70	3.80	3.77	3.90
	(0.64)	(0.64)	(0.54)	(0.58)
Competence	3.65	3.38	3.70	3.59
	(0.62)	(0.47)	(0.55)	(0.51)
Domain	Study 2			
	*Statistics*	*Narrative*		
	*Statistics frame*			
Warmth	3.95	3.97		
	(0.56)	(0.57)		
Competence	3.87	3.54		
	(0.46)	(0.56)		
	*Narrative frame*			
Warmth	3.88	3.91		
	(0.58)	(0.55)		
Competence	3.7	3.52		
	(0.49)	(0.52)		
	*Total*			
Warmth	3.91	3.94		
	(0.57)	(0.56)		
Competence	3.77	3.53		
	(0.48)	(0.54)		

Probing this interaction is complicated by the interdependence of warmth and competence judgments. Prior research has suggested that these constructs have a dynamic relationship in which they mutually influence one another. Furthermore, as they are both assessed with self-report scales, they presumably share common method variance. As a result of these factors, the two have a significant positive correlation (*r* = .35, *p* < .001). Failing to account for this interdependency would therefore lead to a less powerful test with misleading standard errors.

To account for the interrelationship of warmth and competence judgments, we assessed the effect of argument type on each variable controlling for the other using ANCOVA. The effect of argument type was significant for both warmth (controlling for competence), *F* (1, 218) = 7.62, *p* = .006, $\eta_{p}^{2}$ = .03, and competence (controlling for warmth), *F*(1, 218) = 20.38, *p* < .001, $\eta_{p}^{2}$ = .08. In other words, a source using narrative information was seen as significantly warmer (supporting H1) and a source using statistical information was seen as significantly more competent (supporting H2).

These simple effects can be observed in the raw means, and are significant when interdependency is accounted for using ANCOVA. However, it is also true that they do not consistently reach significance when each outcome is examined in isolation. This difference can be attributed to how these two test strategies treat the variance common to warmth and competence. When testing in isolation, the common variance will unavoidably be noisy error variance, leading to inflated standard errors. Under our ANCOVA strategy, the common variance will not be lumped in with the error variance, leading to more accurate standard errors and a more powerful test.

To address RQ1, further analyses were conducted to test whether the effect of argument type on person perception was moderated by individual differences. To aid interpretation, all individual-difference measures were mean-centered ahead of analysis (such that a score of zero represented an average individual). Each DV was predicted from argument type, an individual difference dimension, the other person perception dimension (as a control), and the interaction between argument type and individual difference. The three individual difference variables were analyzed separately, leading to a total of six analyses (two dimensions of person perception and three individual differences). The key test was whether the interaction effect was significant, indicating that the effect of argument type depended on individual differences. The effect failed to reach significance across all three individual differences: need for cognition (*B*s > -0.07, *p*s > .64), need for affect (*B*s < 0.09, *p*s > .39), and transportability (*B*s < 0.10, *p*s > .18). As such, the current study provided no evidence that the effect of argument type on person perception was dependent on individual differences.

### Discussion

As hypothesized, the type of evidence used affected perceptions of the message source: a narrative argument led to greater perceptions of source warmth, and statistical arguments led to greater perceptions of source competence. Even though warmth and competence were significantly correlated, perhaps indicating that participants formed an overall positive or negative impression of the individual, the type of argument used had a distinct influence on the two dimensions. This effect was not moderated by individual differences in need for cognition, need for affect, or transportability.

## Study 1b

We suggest that these effects should be robust across populations. Thus, Study 1b was conducted to replicate the findings of Study 1a in a non-student sample.

### Methods

The methods for Study 1b were largely identical to Study 1a.

#### Participants

Mechanical Turk users (N = 255) participated for a small payment. The sample was majority male (63%) and white (83%). Ages ranged from 18 to 71 (*M* = 31.20, SD = 10.83). Fourteen users (5%) failed the attention check. No changes in significance occurred when these participants were excluded; therefore, all analyses are reported with the full sample.

As this study had the same design and a slightly larger sample, it should be similarly powered. A post-hoc sensitivity analysis confirmed this; the current study was adequate to reliably detect fairly small effect sizes (80% power; f2 = .03, *η*^2^ = .03).

### Results and discussion

As in Study 1a, participants rated a source who used a narrative argument as warmer but less competent than a source who used a statistical argument (see Table 3 and Fig 2). The two DVs were significantly correlated (*r* = .49, *p* < .001), and so were analyzed simultaneously using the same ANOVA design as the previous study. As before, this analysis revealed a significant interaction between rating (warmth vs. competence) and argument type, *F*(1, 241) = 11.34, *p* < .001, $\eta_{p}^{2}$ = .05. Furthermore, an ANCOVA of each DV controlling for the other revealed significant simple effects of argument type for both warmth, *F*(1, 241) = 8.32, *p* < .01, $\eta_{p}^{2}$ = .03, and competence, *F*(1, 235) = 8.34, *p* < .01, $\eta_{p}^{2}$ = .03, supporting H1 and H2.

To address RQ1, the moderating effect of individual differences was tested using a series of regression analyses. As before, the individual difference scores were mean-centered and separate analyses were conducted to assess the effect of each scale (need for cognition, need for affect, transportability) on each person perception dimension (warmth vs. competence). The IVs were the same as in Study 1a. As in Study 1a, these analyses failed to reveal any significant interactions between argument type and individual difference scale: need for cognition (*B*s < .04, *p*s > .41), need for affect (*B*s > -.001, *p*s > .76), or transportability (*B*s < .008, *p*s > .41).

This replication of Study 1a demonstrated that the hypothesized effects are robust not only against the influences of individual differences, but also across populations.

## Study 2

Studies 1a and 1b demonstrate and confirm the basic effects of evidence type on person perception across different populations. They provide clear initial support for the Message-Based Impression Formation effect, that message features can influence perceptions of the source. However, the design of these studies presumes that the target is receiving the source’s advice without any preference for the type of advice that is given. In some contexts, the targets of messages may have preferences for a specific type of evidence (narrative or statistical) for multiple reasons. For example, the topic may seem more appropriate for one evidence type or another, or there may be individual differences in whether individuals prefer stories or facts.

Such preferences for evidence type may moderate the effects demonstrated in Studies 1a and 1b. If a target explicitly states a preference for a particular information type, then a source who provides this information may be seen as more competent (as they are providing the most appropriate information) and / or more warm (as they are more responsive to the target’s preferences). Both effects are equally plausible possibilities given the lack of prior research on this topic.

Thus, in Study 2, we extended the findings of studies 1a and 1b by examining an additional potential moderator: the target’s desired type of evidence.

**RQ2**: Are the effects of evidence type on person perception moderated by the target’s desired type of evidence?

We also added subjective numeracy as an additional individual difference in Study 2, as comfort with numerical information might logically moderate appraisals of statistical evidence.

### Methods

The methods for Study 2 were largely identical to Studies 1a and 1b with the addition of frames to convey the target’s preferred type of evidence. As need for affect had resulted in consistent null findings across Studies 1a and 1b, it was dropped. In its place, subjective numeracy [29] was added.

#### Participants

Mechanical Turk users (N = 224) participated in this study for a small payment. Five participants (2%) failed the attention check. No changes in significance occurred when these participants were excluded; therefore, all analyses are reported with the full sample. The sample was majority male (67%) and white (81%).

For this study, we were aiming to a) replicate the two-way interaction between message type and personality dimension from the previous studies and b) test whether this effect is moderated by the targets’ expressed preferences (i.e., a three-way interaction). We conducted sensitivity analyses for these two key tests. For two-way interactions, our sample is adequate to reliably detect reasonably small effects (80% power; f2 = .04, *η*^2^ = .04). Three-way interactions are inevitably going to be harder to detect, but the current sample is still sufficient to consistently detect fairly modest effects (80% power; f2 = .05, *η*^2^ = .05).

#### Frames

The target-preference frames consisted of a single sentence at the end of the vignette’s introduction paragraph: “He’s particularly interested in “hearing about the customer experience at First City Bank” [narrative] or “getting the facts about First City Bank” [statistical].

### Results

First, to assess the effects of the manipulations, the results were analyzed using a mixed ANOVA. The analysis followed a 2 (Argument type: Narrative vs. Statistics) x 2 (Framing: Narrative vs. Statistics) x 2 (Person perception dimension: Warmth vs. Competence) design, with repeated measures on the final factor. The means are given in Table 3.

The interaction between argument type and person perception dimension was replicated, *F*(1, 219) = 16.81, *p* < .001, $\eta_{p}^{2}$ = .07. This interaction was probed using an ANCOVA of each DV controlling for the other. These subsequent analyses revealed significant simple effects of argument type for both warmth, *F*(1, 219) = 8.38, *p* < .01, $\eta_{p}^{2}$ = .04, and competence, *F*(1, 219) = 22.60, *p* < .001, $\eta_{p}^{2}$ = .09.

There was no evidence that the effects of argument type were moderated by the framing manipulation. There was no significant effect of frame, nor did it show any significant two-or three- way interaction with the other factors, *F*s(1, 219) < 1.74, *p*s > .18, $\eta_{p}^{2}s$ < .01.

Second, regression analyses were used to examine whether the effects of argument type were moderated by individual differences. Individual difference scores were mean-centered, and separate analyses were conducted for each scale and person perception dimension. The analyses were similar to those in the previous study, but also included the framing manipulation and its interaction with argument type. There was no significant interaction between individual difference and argument type for need for cognition (*B*s < -0.01, *p*s > .67), subjective numeracy (*B*s < 0.05, *p*s > .15), or transportability (*B*s < 0.15, *p*s > 0.16).

### Discussion

Study 2 provided further evidence for the basic effect, and the primary findings were not affected by the framing manipulation. Regardless of the target’s expressed preferences, sources were seen as more warm when they provided narrative information and more competent when they provided statistical information.

It is possible that the frame did not properly convey the target’s desire for narrative or statistical evidence; additionally, it is possible that participants thought the source might be unaware of the target’s preferences (as the frame was not expressed to the source in the vignette), and did not judge the source accordingly. However, we suggest that these findings may instead represent the general robustness of this effect: statistical and narrative arguments may be strongly enough identified with competence and warmth to overcome individual preference. Null findings for individual differences throughout support this interpretation.

## Study 3a

Studies 1–2 have established a replicable effect of persuasive evidence type on person perception: across different samples, narratives are linked to warmth and statistics are linked to competence. This effect is robust against individual differences or framing.

These results, however, do not test the full M-BIF pathway from message to perceptions to outcomes. If evidence type provides real information about an individual’s warmth and competence, this information should have some relevance for decisions about relationships and social interactions–a warm person may make a better friend, a competent person a better coworker. Given narrative or statistical evidence in response to a question, do individuals use this information to make strategic judgments about future interactions?

To test this question, Studies 3a and 3b examine the effects of evidence type on partner preference across different types of task. In Study 3a, participants were assigned to a partner who has provided narrative or statistical evidence and asked to choose between working with this partner against another team in a competitive or cooperative task; this design is flipped in Study 3b, where participants were assigned to a competitive or cooperative task and asked to choose between partners who have written narrative or statistical evidence.

Across both studies, we hypothesized a link between the provision of statistical evidence and the competitive task and a link between the provision of narrative evidence and the cooperative task. In other words, if asked to choose between two different tasks that require different aptitudes in a partner or two different partners with presumably different skillsets, participants will use what they have learned from their partner’s choice of evidence type to strategically pick the task that maximizes their chance of success.

To conduct this study, narrative and statistical arguments of equal quality that could have conceivably been written by an interaction partner were required. In Studies 3a and 3b, we chose a different domain to investigate: rather than financial advice, these studies examined arguments about learning. Additionally, the design required two tasks: a cooperative task seen as strongly suited to perform with a warm partner alongside another team, and a competitive task seen as strongly suited to perform with a competent partner against another team.

### Method

Participants were asked to imagine they would be performing a task online with an interaction partner whom they had never met. Participants then read a narrative or statistical argument on the topic of active learning, ostensibly written by their partner. The quality of the argument and perceptions of the argument’s author were then rated as in previous studies. Participants lastly chose between two tasks they could complete with their partner, and assessed these tasks on various dimensions.

#### Participants

Mechanical Turk users (N = 104) participated for a small payment. The sample was majority male (56%) and white (84%). Ages ranged from 18 to 70 (*M* = 29.26, SD = 9.91). A sensitivity analysis showed this sample is adequately powered to detect a Cohen’s w of 0.27 (80% power chi squared test on a 2x2 contingency table).

As an attention check, participants were asked “What topic did the conversation you read discuss?” Participants passed this check if they provided an answer relating to active learning, education, or the value of working with others in education. Eight participants (5%) failed. All analyses are reported with all participants included; where exclusion of these participants changed the significance of the results, analyses are also reported with the smaller sample.

### Materials

#### Task choice and assessment

The measure of task choice asked participants if they would rather “complete a simple competitive task against another team?” or “chat with another team about your shared interests and hobbies?” Both tasks involved working with the argument’s author as part of one team, to ensure that participants would view the argument’s author as a colleague rather than a direct competitor.

Participants were also asked to rate the extent to which they would like to complete each task, both in general and with their specific partner. Participants also rated how much each task required a competent partner and a friendly partner. A final choice measure asked participants to decide if they would prefer a friendly or competent partner for each task.

#### Narrative argument

The narrative argument read as follows: **“**I think active learning is a really great way to help people succeed in the classroom. One of my friends was in a high school science class that used active learning–working in teams together to solve problems in class time, discussing topics rather than just listening to lectures. She told me she learned a lot more in those classes, because it was easier to pay attention and she thought a lot more deeply about the material–and enjoyed it a lot more. She said it was fun getting a chance to work more closely with her classmates and hearing their thoughts on the material. And, at the end of the year, she did a lot better on both her report card and standardized tests. Teachers should definitely try to include more active learning in their classroom planning.”

#### Statistical argument

The statistical argument read as follows: “I think active learning is a tested and proven way to help people succeed in the classroom. Research shows that active learning techniques like using class time to have students work together on problems instead of listening to lectures result in as much as ten percent gains in long-term memory for information. By making it easier for students to pay attention and requiring more thought, they also improve standardized test scores–up to twenty percent–and grades by as much as a letter and a half. Student satisfaction is also significantly improved in every way, from interest to satisfaction to self-reported learning, over more traditional lecture classes. Teachers should definitely try to include more active learning in their classroom planning. “

### Results

#### Argument assessment

The narrative argument (M = 4.70, SD = 1.55) and statistical argument (M = 5.13, SD = 1.21) did not differ significantly in quality, *t*(101) = -1.56, *p* > 0.05, *d* = 0.31. Additionally, both arguments were significantly higher on their intended character; see Tables 1 and 2.

#### Task choice

Across all conditions overall, participants showed a preference for the competitive task, χ^2^(1) = 7.54, *p* < 0.01; 63% competitive task choice. However, when asked to imagine completing the task with the argument author, participants were more likely to choose the cooperative task (52%) in the narrative condition than in the statistical condition (32%), χ^2^(1) = 4.56, *p* < 0.05. With participants who failed the attention check dropped, this effect is marginal, χ^2^(1) = 3.04, *p* = 0.08.

#### Person perception

The data for person perception was analyzed using a 2 (argument type: statistical vs. narrative) x 2 (rating: warmth vs. competence) ANOVA with repeated measures on the final factor. This analysis revealed a significant interaction between argument type and rating, *F*(1, 101) = 23.85, *p* < .001, $\eta_{p}^{2}$ = 0.03. To probe this interaction, the effect of argument type on each variable controlling for the other was assessed using ANCOVA. As hypothesized, competence was significantly higher in the statistical condition (controlling for warmth; *F*(1,100) = 19.54, *p* < 0.001, $\eta_{p}^{2}$ = 0.16); warmth was significantly higher in the narrative condition (controlling for competence; *F*(1,100) = 18.24, *p* < 0.001, $\eta_{p}^{2}$ = 0.15).

### Discussion

Study 3a robustly replicated prior findings about the influence of argument type on person perception. Its results also provide initial evidence in support of the hypothesis that task choice may depend on evidence type, supporting the M-BIF message-perception-outcome pathway. However, this study involved hypothetical decisions: it simply asked participants to imagine they might be engaging in the activity with the person who wrote the passage. Moreover, participants tended to prefer the competitive task across both conditions, making it difficult to observe the intended effect.

## Study 3b

Study 3b thus included the same measures as Study 3a but used a cover story to support the idea of direct interaction between the participant and the author of the narrative or statistical argument. That is, participants were told that they would be working with the partner they chose. This allowed for the assessment of actual behavior, as participants believed they were making a meaningful choice that would affect their experience in the next part of the study.

In order to eliminate the possible issue of participants’ preference for the competitive task, the variables of partner assignment and task choice were flipped; in Study 3b, participants were instead assigned to a task and asked to choose a partner after reading both vignettes.

### Methods

#### Participants

Undergraduates (N = 203) participated for course credit. The sample was majority female (74%) and white (68%). Ages ranged from 18 to 29 (*M* = 18.56, SD = 1.17). A sensitivity analysis showed this sample is adequately powered to detect an odds ratio of 2.26 (80% power in a 2-tailed logistic regression, assuming a base rate of 0.5).

For this study, participants completed suspicion checks as well as attention checks. Suspicion was coded by asking participants what, if anything, we had told them might not be true. Only nine participants (4%) doubted the existence of a partner on their own; however, when explicitly asked if they suspected they would not be working with a partner, 73 participants (36%) answered in the affirmative. It is likely many of those participants did not truly suspect anything until they were directly prompted by the question, and omitting all 73 participants from the sample does not alter the significance of the basic partner choice by task assignment finding. Additionally, only three (1%) participants failed an attention check identical to Study 3b’s, and their exclusion does not alter the significance of results. As such, all analyses are reported with all participants included.

#### Procedure

Participants in Study 3b were brought into a laboratory and told they were participating in a study where some participants had been chosen as writers and others as readers. The writers had been asked to write a passage on the topic of active learning, and had agreed to be available at a pre-chosen time to be matched with a reader to complete a task. All participants actually viewed instructions for the “reader” condition, and were then randomly assigned to either the competitive task or the cooperative task.

After assignment to a task, participants were provided both the narrative and statistical arguments, characterized as the output of two potential partners from the writing session. The participant then chose a specific partner to work with on their assigned task. Participants then provided measures of person perception, individual differences, and demographics.

### Measures

#### Arguments and partner choice

The arguments and tasks validated in Study 3a were used for Study 3b. The tasks were described with slightly more information, as follows: “a simple competitive task against another pair of participants. In this activity, your goal is to outperform the other pair of participants” or “a chat with another pair of participants about your shared interests. In this activity, your goal is to get to know the other participants.”

Participants were asked to choose one of the two partners to work with on their assigned task after reading both passages. Participants were also asked, on a 1–7 scale, how much they would wish to work with each partner on their task.

#### Other measures

Person perception, argument quality, the narrative or statistical nature of the task, transportability, need for cognition, and demographics were all measured as in prior studies.

### Results

#### Person perception

As all participants rated both passages on warmth and competence, person perception variables were compared across passages by a 2 (domain of person perception: warmth or competence) by 2 (evidence type: narrative or statistical) ANOVA with repeated measures on both factors. The interaction between domain of person perception and evidence type was significant, *F*(1, 201) = 836.28, *p* < 0.0001, confirming that participants rated the authors differently on warmth and competence depending on whether the passage was narrative or statistical. As expected, the narrative author was rated as warmer (mean difference = 1.03, SD = 0.78, *t*(198) = 18.75, *p* < 0.001), and the statistical author was rated as more competent (mean difference = 1.09, SD = 0.66, *t*(195) = -22.74, *p* < 0.001).

#### Partner choice by task

**Fig 3:**
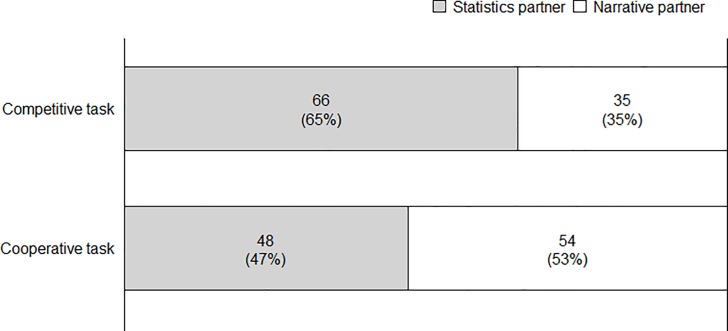
Partner choice (shown as percentage) by task assignment in Study 3b.

Logistic regression was conducted to examine the effect of task assignment on partner choice, coding choice of the statistical partner as 0 and choice of the narrative partner as 1. Partner choice was significantly dependent on task assignment, χ^2^ = 6.80, *p* < 0.01 (see Fig 3).

52.9% of the participants assigned to the cooperative task chose the narrative partner, as opposed to only 34.6% of those assigned to the competitive task (an odds ratio of 2.12).

#### Partner choice by task and perception

An additional analysis included perceptions of warmth and competence of both partners in the regression. The perceptions of each partner do not occur in a vacuum; the task that participants engage in is inherently comparative. As such, the warmth and competence variables were coded for this analysis as perceptions of the statistical partner minus perceptions of the narrative partner–positive values indicating more positive perceptions of the statistical partner, while negative values indicate more positive perceptions of the narrative partner. This model significantly predicted choosing the statistical partner, χ^2^ (3) = 54.12, *p* < 0.001, with task assignment (odds ratio 2.74; χ^2^ (1) = 9.31, *p* = 0.002), greater competence of the statistical partner (odds ratio 3.79; χ^2^ (1) = 21.73, *p* < 0.0001), and greater warmth of the statistical partner (odds ratio 3.35; χ^2^ (1) = 21.17, *p* < 0.0001), all serving as significant factors. In other words, individuals who saw the statistical partner as more competent and more warm than the narrative partner were more likely to choose the statistical partner, but so were individuals assigned to the competitive task.

A third model added the interaction between the task and the person perception variables to model the hypothesis that competence differences between partners would be more predictive for those assigned to the competitive task, whereas warmth differences between partners would be more predictive for those assigned to the cooperative task. This model was significant overall in predicting partner choice (χ^2^ = 48.80, *p* < 0.0001). However, the effect of the task was no longer significant with the other variables included (χ^2^ = 1.37, *p* > 0.05); in addition, interactions between the task and warmth difference between partners (χ^2^ = 1.36, *p* > 0.05) and the task and competence difference between partners (χ^2^ = 1.32, *p* > 0.05) were not significant. However, both the warmth difference (χ^2^ = 12.34, *p* < 0.001) and the competence difference (χ^2^ = 12.92, *p* < 0.001) between partners remained significant predictors of choice, as predicted.

Interactions between task assignment and individual differences were included separately in each analysis, but did not predict partner choice in any analysis.

#### Desire to work with partners by task

To further examine the role of task assignment in predicting partner choice, a 2 (task assignment: cooperative vs. competitive) by 2 (partner being rated: narrative or statistical) ANOVA with repeated measures on the final factor was conducted, predicting desire to work with both partners. As hypothesized, the interaction between task assignment and partner being rated was significant, *F*(1,198) = 6.27, *p* = 0.01, $\eta_{p}^{2}$ = 0.03, suggesting that task assignment affected the partners’ desirability differently. This effect was probed using univariate ANCOVAs, which examined desire to work with each partner while controlling for desire to work with the other partner. These analyses revealed a significant effect of task assignment on desire to work with the narrative partner, *F*(2, 197) = 6.20, *p* = 0.01, $\eta_{p}^{2}$ = 0.03, but no evidence for an effect of task assignment on desire to work with the statistical partner, *F*(2, 197) = 0.93, *p* = .34, $\eta_{p}^{2}$ < 0.00.

### Discussion

Study 3b supports our hypothesis that the role of evidence type goes beyond person perception–it has the potential to drive real-world choices about the individuals with whom we choose to interact in order to maximize our success. Those who expect competition against others are more likely to select partners who use statistical evidence for their perceived competence, while those who expect social interaction are more likely to seek out partners who use narrative evidence for their perceived warmth.

Follow-up analyses, however, suggest that the process may be slightly more nuanced than a simple link between task type and partner choice. Regardless of task assignment, participants were more likely to choose a given partner based on both higher warmth and higher competence. Additionally, the statistical partner was chosen more frequently and appeared to be equally desired across different tasks, whereas the narrative partner’s appeal was task-dependent.

This finding was not hypothesized and, at first glance, might seem to contradict existing literature on the primary importance of warmth in choice of interaction partners [35]. If individuals primarily select their interaction partners on the basis of warmth, then one might expect an opposite pattern from the one we observed: a statistical partner should only be valued in the competitive task, while a narrative partner should be valuable in either task. However, several differences between the paradigm in [35] and that utilized here may explain the discrepancy.

Most significantly, Cottrell et al. [35] do not define their terms as we do here–while ‘trustworthiness’ may be a direct proxy for warmth, their research lacks any direct proxy for competence, including only ‘intelligence’ as the closest concept. They also focus on individuals with whom one is either interdependent (such as a teammate or romantic partner) or casually encountering in a social situation (such as an acquaintance)–in contrast, our design focuses on a single interaction explicitly oriented toward task completion. In the closest such situation [35] tested, that of choosing ideal traits for a coworker, intelligence was chosen as the most important, while trustworthiness came second–a finding that neatly dovetails with our results.

## General discussion

Across three studies, narrative evidence increases perceptions of source warmth, while statistical evidence increases perceptions of source competence. Though the effect sizes are small, these results are robust against the influences of framing and individual differences and have replicated across different samples and designs, and with messages about different topics (financial advice; education). Additionally, individuals differentially choose interaction partners in task-based scenarios based on evidence type, implying that these effects go beyond the realm of perception into behavior.

These results extend research in multiple ways. First, by bringing together research on persuasion with work on person perception, these studies and the Message-Based Impression Formation effect highlight an important and understudied consequence of interpersonal communication. Persuasive messages not only convey content, but also influence individuals’ impressions of the message source. The research reported here provides an initial demonstration of the links between message features, person perception, and outcomes. We have focused here on one specific message feature, but the links that we have drawn here between messages and impression formation can help guide and encourage future research more broadly on this relatively neglected topic.

Second, these studies illuminate the effects that using different types of evidence can have on interpersonal interactions. Everyday persuasive contexts may have considerable implications for social life, particularly during relationship formation–perceptions of warmth and competence are likely to influence who we choose to approach for closer relationships. Studies 3a and 3b also speak to the importance of these results beyond the initial context of impression formation. If the use of narrative or statistical information in these interactions can drive not only person perception but also the choice of social partners for different activities in the future, the type of evidence we bring to bear when communicating with others interpersonally may have considerable impact on the course of our relationships. Those who wish to flourish in the social sphere may benefit from the use of narratives, while those who wish to flourish in professional matters may find statistical persuasion more valuable.

Furthermore, there are clear practical implications of these findings for a range of interpersonal communication settings, including political and business communications. Communicators may not wish to only consider which type of evidence will be more persuasive, but also the impressions conveyed by their choice. For example, individuals who are already high in expertise (such as scientists) may benefit from the use of narratives to bolster their warmth; similarly, individuals who are high in liking or attractiveness may benefit from the use of statistical information to bolster their competence. Such source perceptions may then influence the effectiveness of the communication attempt, above and beyond the message content. A scientist who uses narratives and is therefore perceived more warmly may evoke less reactance than one perceived to be cold and distant, which may be important when communicating about potentially divisive issues such as climate change [36].

The current studies have focused primarily on positive outcomes, such that the messages created generally good impressions of warmth and competence. However, persuasive messages may also have negative effects on interpersonal perceptions. Future research might examine the conditions under which messages might backfire. For example, there is some evidence that a poorly-told story (such as one that rambles and diverges from the main point) can reduce the storyteller’s perceived attractiveness [37]. Persuasion research suggests that weak arguments can reduce persuasion, at least under conditions of high elaboration; it remains an open question whether poor arguments also lead to more negative interpersonal perceptions. An unconvincing argument may make the speaker appear less competent, but perhaps only to attentive (high elaboration) audiences.

Additionally, although the current paper focuses on the type of evidence used in a communication, studies of Message-Based Impression Formation effects can be expanded to encompass other forms of information (message features) that may be conveyed by messages. First, and perhaps most obviously, individuals form impressions based on the message content. For example, if a person praises conservative policies or candidates, observers will infer that the person is politically conservative. Second, the quality of a message may influence perception of a source: teachers may form quite different impressions of students who submit sloppy papers versus well-written ones. Additionally, future research may identify other features of messages may also create different impressions.

### Future directions

Future research should identify moderators of these effects. One possibility is the pre-existing relationship between the target and source. It is plausible that argument type is more salient when the target does not know the source well. Second, there may also be situational or individual differences in preferences for narrative or statistical information. Our framing manipulation did not demonstrate such effects; nonetheless, it is possible that explicit statements of a target’s preference (e.g., “don’t bore me with numbers” or “just the facts, please”) may also influence how the source is viewed when they provide evidence that either matches or contradicts the expected type. Our vignettes also used male communicators in the scenario studies, although the gender of the partner was not specified in the behavioral studies (3a and 3b). Given established links between stereotypical views of women and warmth, and men and competence [38], different results might arise with communicators of different genders. Finally, future research should explore what happens when communicators combine both types of evidence.

Future research should also consider the further relational implications of Studies 3a-3b. Our design did not include a genuine interaction between participants and individuals they believed were the authors of the passages; it remains to be seen if participants might actually interact differently with narrative or statistical persuaders. The influence of evidence type should also be studied in other fields where individuals must be selected for certain roles, such as hiring or task assignment in the professional world.

While the choice between narrative and statistical evidence has often been studied solely in the context of attitudinal change, this research suggests it may have deeper implications for our everyday lives. When individuals judge the message, they may also be judging the messenger, and these judgments may be consequential for future social interaction.
